# Analysis of Hypoxic and Hypercapnic Ventilatory Response in Healthy Volunteers

**DOI:** 10.1371/journal.pone.0168930

**Published:** 2017-01-03

**Authors:** Shmuel Goldberg, Hanna Maria Ollila, Ling Lin, Husham Sharifi, Tom Rico, Olivier Andlauer, Adi Aran, Efrat Bloomrosen, Juliette Faraco, Han Fang, Emmanuel Mignot

**Affiliations:** 1 Pediatric Pulmonology Unit, Shaare Zedek Medical Center, Hebrew University, School of Medicine, Jerusalem, Israel; 2 Stanford University Center for Sleep Sciences, Palo Alto, CA, United States of America; 3 East London NHS Foundation Trust, Newham Centre for Mental Health, London, United Kingdom; 4 Neuropediatric unit, Shaare Zedek Medical Center, Hebrew University, School of Medicine, Jerusalem, Israel; 5 Department of Family Medicine, Hebrew University and Clalit Health Services, Jerusalem, Israel; 6 Department of Pulmonary Medicine, Peking University People's Hospital, Beijing, China; Charité - Universitätsmedizin Berlin, GERMANY

## Abstract

**Introduction:**

A previous study has suggested that the Human Leukocyte Antigen (HLA) allele DQB1*06:02 affects hypoxic ventilatory response (HVR) but not hypercapnic ventilatory response (HCVR) in an Asian population. The current study evaluated the relationship in Caucasians and Asians. In addition we assessed whether gender or polymorphisms in genes participating in the control of breathing affect HVR and HCVR.

**Methods:**

A re-breathing system was used to measure HVR and HCVR in 551 young adults (56.8% Caucasians, 30% Asians). HLA-DQB1*06:02 and tagged polymorphisms and coding variants in genes participating in breathing (PHOX2B, GPR4 and TASK2/KCNK5) were analyzed. The associations between HVR/HCVR and HLA-DQB1*06:02, genetic polymorphisms, and gender were evaluated using ANOVA or frequentist association testing with SNPTEST.

**Results:**

HVR and gender are strongly correlated. HCVR and gender are not. Mean HVR in women was 0.276±0.168 (liter/minute/%SpO2) compared to 0.429±0.266 (liter/minute/%SpO2) in men, p<0.001 (55.4% higher HVR in men). Women had lower baseline minute ventilation (8.08±2.36 l/m vs. 10.00±3.43l/m, p<0.001), higher SpO2 (98.0±1.3% vs. 96.6±1.7%, p<0.001), and lower EtCO2 (4.65±0.68% vs. 4.82±1.02%, p = 0.025). One hundred and two (18.5%) of the participants had HLA-DQB1*06:02. No association was seen between HLA-DQB1*06:02 and HVR or HCVR. Genetic analysis revealed point wise, uncorrected significant associations between two TASK2/KCNK5 variants (rs2815118 and rs150380866) and HCVR.

**Conclusions:**

This is the largest study to date reporting the relationship between gender and HVR/ HCVR and the first study assessing the association between genetic polymorphisms in humans and HVR/HCVR. The data suggest that gender has a large effect on hypoxic breathing response.

## Introduction

Mammals respond to hypercapnia and hypoxia by increasing minute ventilation (Ve) to maintain a neutral balance of acid and base. Hypercapnia is the main driver of the ventilatory response and is likely mediated by the retrotrapezoid nucleus (RTN) [1], which is located in the ventral medullary surface and projects neurons to the respiratory rhythm generator. The consequent hypercapnic ventilatory response (HCVR) is expressed as the change in Ve per change in CO2 at the end of exhalation (ΔVe /ΔEtCO2). In contrast hypoxic ventilatory response (HVR) arises from peripheral chemoreceptors that sense the partial pressure of oxygen and are located mainly at the bifurcation of the common carotid arteries. It is expressed as the change in Ve (liter/minute) per change in percentage oxygen saturation (ΔVe /ΔSpO2).

There are differences between individuals in these ventilatory responses, some of which are believed to be genetic [2]. A recent study reported the surprising finding that Chinese carriers of the human leukocyte antigen (HLA)-DQB1*06:02 have reduced HVR [3]. Almost all narcolepsy patients are carriers of this HLA allele [4], and abnormal HVR may influence the clinical presentation of narcolepsy. In the current study we tried to replicate this finding.

Other genes that may give rise to differences in control of breathing, especially during sleep, include paired-like homeobox 2B (PHOX2B), GPR4 and KCNK5/TASK2. Located on chromosome 4, the PHOX2B gene is involved in neurogenesis and codes for a homeodomain transcription factor. In mice PHOX2B mutations abort RTN development and cause a deficit in HCVR [5]. PHOX2B contains a repeat sequence of 20 alanines in exon 3. Such a polyalanine tract correlates with Congenital Central Hypoventilation Syndrome, an impairment of autonomic ventilatory control with preservation of voluntary ventilatory control. It manifests most prominently in sleep, when the body demonstrates a reduced response to hypoxia and hypercapnia [6].

GPR4 and KCNK5/TASK2 are two genes recently identified as critical to CO2-stimulated breathing [7,8]. Genetic deletion of GPR4 in the RTN shows multiple effects on breathing, including disruption of acidosis-dependent activation of RTN neurons, increased apnea frequency, and blunted ventilatory response to CO2. Additional elimination of TASK-2 (K(2P)5), a pH-sensitive K(+) channel expressed in RTN neurons, essentially abolishes the ventilatory response to CO2 [8]. A second goal of the current study was to investigate the influence of single nucleotide polymorphisms (SNPs) in the PHOX2B, GPR4 and KCNK5/TASK2 genes on HVR and HCVR.

Due to the size of the cohort, we were also able to gather epidemiological data to assess for associations between age, gender, ethnic origin, and body surface area (BSA) with response to hypoxia and hypercapnia.

## Methods

### Ethical approval

Written informed consent was obtained in accordance with Stanford University from all subjects. The research protocol was approved by the Institutional Review Board (IRB) Panel on Medical Human Subjects at Stanford University.

### Participants

Participants (N = 551) were healthy volunteers (38.7% men, mean age 24 years old, Table 1), mainly students and staff of Stanford University and Stanford Medical Center. Exclusion criteria included pregnancy, current smoking or a past history of smoking of at least 1 pack per day for 1 year, a respiratory tract infection in the past month, asthma with any symptoms in the last 3 years, history of prolonged mechanical ventilation, use of supplemental oxygen for any reason, and any other chronic pulmonary disease. We also excluded volunteers with cardiovascular disease such as ischemic cardiomyopathy, prior acute coronary syndrome, carditis of any form, prior cardiac surgery, valvular stenosis, valvular regurgitation, and arrhythmias.

**Table 1:** Baseline epidemiologic and physiologic data.

Variable	All (n = 551)	Men (n = 210)	Women (n = 332)	p value*
Age (years)	24.1 ± 7.3 †	23.9 ± 7.0	24.2 ± 7.5	0.71
Ethnicity, n (%)				0.683
Caucasian	313 (56.8)	124 (59.0)	189 (56.9)	
Asian	121 (30.0)	44 (21.0)	77 (23.2)	
Other	117 (21.2)	42 (20)	66 (19.9)	
Body Surface Area (BSA) (m^2^)	1.81 ± 0.23	1.97 ± 0.22	1.71 ± 0.17	<0.001
Body Mass Index (BMI) (kg/m^2^)	23.26 ± 3.79	24.11 ± 4.12	22.72 ± 3.45	<0.001
HLA-DQB1*0602 positive, n (%) §	102 (19.0)	41 (19.6)	61 (18.5)	0.729
Baselinerespiratory rate (RR) (breaths/min)	13.0 ± 4.5	12.4 ± 4.6	13.4 ± 4.1	0.007
Baseline minute ventilation (MV) (liters/min)	8.86 ± 3.05	10.00 ± 3.43	8.08 ± 2.36	<0.001
RR/BSA (breaths/min/m^2^)	7.3 ± 2.9	6.4 ± 2.8	7.9 ± 2.8	<0.001
MV/BSA (liters/min/m^2^)	4.91 ± 1.61	5.14 ± 1.77	4.78 ± 1.46	0.016
Baseline SpO_2_ (%) unadjusted	97.4 ± 1.6	96.6 ± 1.7	98.0 ± 1.3	<0.001
Baseline SpO_2_ (%) adjusted				<0.001
Baseline EtCO_2_ (%) unadjusted	4.71 ± 0.86	4.82 ± 1.02	4.65 ± 0.68	0.025
Baseline EtCO_2_ (%) adjusted				0.012

* p value is assessed as men versus women.

† Mean age from 542 participants, as data for age were missing from 9 participants.

‡ Data for gender were missing from 9 participants.

§ A total of 538 participants were tested for HLA-DQB1*0602, of whom 209 were men and 329 were women.

¶ Adjusted for age, ethnicity and BMI.

### Measurements of the respiratory responses to hypoxia and hypercapnia

Respiratory responses to both hypoxia and hypercapnia were measured using a re-breathing system, which has been widely employed after first being described by Read [9]. The same system was used by Han in their study of HLA DQB1 and control of breathing [3]. HVR tests were stopped when a patient reached SpO2 of 75%. HCVR tests were stopped when a patient reached EtCO2 of 7.8%.

The breathing system in this study comprises a closed loop of pipes attached to a facemask that allows control of inspired gases and measurement of end tidal CO2 (EtCO2), respiratory rate, tidal volume, and SpO2. Studies were started between 6:49 AM and 5:29 PM, with 80% of them between 8AM and 1PM. All studies were started after 5 minutes of rest in a sitting position. SpO2, EtCO2, and minute ventilation were recorded in 10-second intervals during the rest time. Each patient’s baseline was the mean of measurements after stabilization of the signal. For assessing response to hypoxia, a poikilocapnic study was done. Participants re-breathed exhaled air that was filtered via a CO2 absorbent (soda lime) to prevent CO2 accumulation. The tests were stopped when the SpO2 reached 75%. Changes in minute ventilation, EtCO2, and SpO2 were recorded. For testing the respiratory response to hypercapnia, participants breathed through the same circuit but without CO2 absorbent. A reservoir bag, filled with 5–7 liters of gas containing 7% CO2, 40% oxygen, and the balance N2, was connected to the mask through a one-way valve that allowed inhalation only. There was thereby a constant increase in inhaled CO2 with no concomitant decrease in inspiratory O2. The tests were stopped when EtCO2 reached 7.8%. The changes in minute ventilation and EtCO2 were recorded using an Ohmeda 5250 RGM multiple medical gas monitor (Ohmeda, Madison, WI, USA). Data was recorded in 10 second intervals.

Reliable HVR tests were obtained in 427 participants. An additional 117 tests were considered unreliable and were not analyzed. Of these, 79 tests were excluded due to a leak from the mask or another suspected technical fault. Thirty-three tests were excluded as a result of the patient not being able to reach completion. Seven of the participants did not perform the test. A total of 369 participants completed the HCVR test. An additional 134 studies were unreliable and therefore not analyzed. As with the hypoxia arm, a group (116 participants) was excluded due to a leak from the mask or another suspected technical fault. Twenty-seven participants in the HCVR arm could not complete the test and were excluded.

### Calculation of the respiratory responses to hypoxia and hypercapnia

The study was conducted in two steps. In the first step we performed a preliminary analysis with a subgroup of 28 participants included in respiratory response studies on different days. The aim was to determine the most reproducible calculation methods for HVR and HCVR. The second step constituted the main study, in which we used the most reproducible methods for assessment of HVR and HCVR in the whole study population.

**Fig 1:**
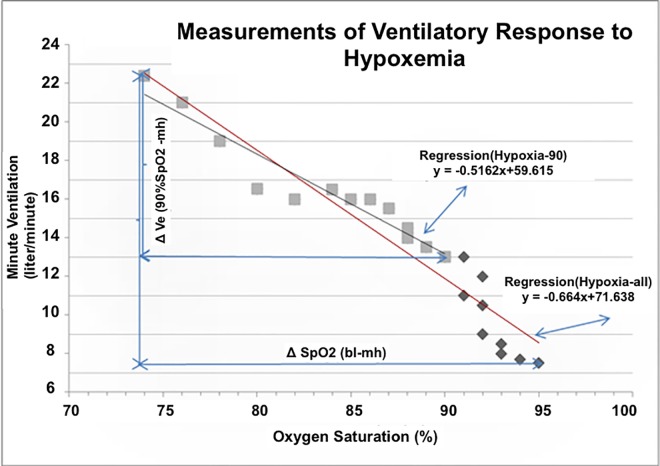
Measurement of ventilatory response to hypoxemia. Illustration of 4 metrics to calculate ventilatory response to hypoxemia (HVR): Hypoxia-all, Hypoxia-90, Regression _(Hypoxia-all_) and Regression _(Hypoxia-90_). The first two variables were calculated by taking the increase in minute ventilation (ΔV_e_) between baseline and maximal hypoxemia (mh) and dividing it by the corresponding change in SpO_2_ (ΔSpO_2_). Baseline minute ventilation differed for Hypoxia-all and Hypoxia-90. In Hypoxia-all baseline minute ventilation was the minute ventilation at the beginning of the study (bl). In Hypoxia-90 baseline minute ventilation was the minute ventilation measured at 90% saturation (90%SpO_2_). Hypoxia-all was calculated using the equation
$$Hypoxia-all\;HVR=\frac{\Delta Ve(bl-mh)}{\Delta SpO2(bl-mh)}$$
Whereas, Hypoxia-90 was calculated using the equation
$$Hypoxia-90\;HVR=\frac{\Delta Ve(90\%-mh)}{\Delta SpO2(90-mh)}$$
Linear regression was applied to the measurements. Regression _(Hypoxia-all_) used the regression line of all measurements. Regression _(Hypoxia-90_) used only measurements done when SpO_2_ was 90% or lower.

In the preliminary analysis we evaluated 4 different methods to calculate HVR with the following variables: Hypoxia-all, Hypoxia-90, Regression (Hypoxia-all), and Regression (Hypoxia-90) (Fig 1). The first two variables were calculated by dividing the difference in minute ventilation (ΔVe) at a prespecified baseline (bl) and at maximal hypoxemia (mh) by the corresponding difference in SpO2 (ΔSpO2) at baseline and at maximal hypoxemia. The baseline for Hypoxia-all was the beginning of the study. The baseline for Hypoxia-90 was the point at which SpO2 reached 90%. HVR at Hypoxia-all and at Hypoxia-90 is therefore described by the following equations:

$$Hypoxia-all\;HVR=\frac{\Delta Ve(bl-mh)}{\Delta SpO2(bl-mh)}$$

$$Hypoxia-90\;HVR=\frac{\Delta Ve(90\%-mh)}{\Delta SpO2(90-mh)}$$

Assessment of respiratory parameters at rest and at SpO2 of 90% was done to ensure that subsequent changes in minute ventilation and SpO2 represented sustained physiological adaptations and not a transient response. The other two variables were calculated using the regression lines of the measurements. Regression (Hypoxia-all) used the regression line of all measurements; whereas, Regression (Hypoxia-90) used only measurements done when SpO2 was 90% or lower.

**Fig 2:**
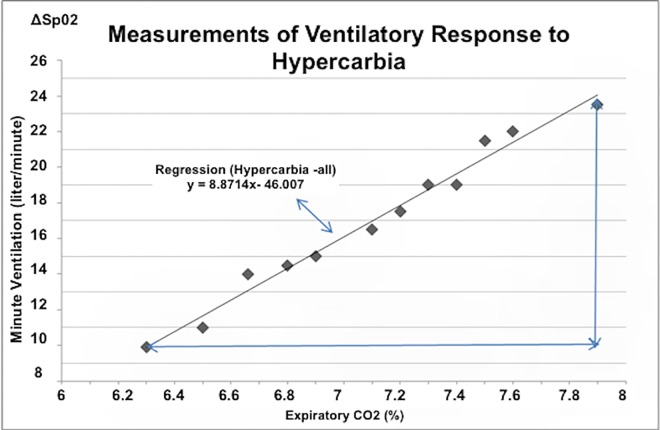
Measurement of ventilatory response to hypercapnia. Illustration of 2 different methods to calculate the ventilatory response to hypercapnia (HCVR) using the following 2 variables: Hypercapnia-all and Regression _(Hypercapnia-all)_. Hypercapnia-all is the change in V_e_ (ΔV_e_) between baseline and the end of the study, divided by the reciprocal change in EtCO_2_ (ΔEtCO_2_) in the following form: HCVR = ΔV_e_/ΔCO_2_. Regression _(Hypercapnia-all)_ is the slope of regression line of all measurements.

In the preliminary step we evaluated 2 different methods to calculate HCVR (Fig 2): hypercapnia-all and Regression (hypercapnia-all). Hypercapnia-all is calculated by dividing the difference in Ve (ΔVe) between baseline and the end of the study by the corresponding difference in EtCO2 (ΔEtCO2): *HCVR* = Δ*Ve*/Δ*CO*2. Regression (hypercapnia-all) is the slope of the regression line of all measurements of respiratory parameters, as displayed in Table 2.

**Table 2:** Correlation (R- value) between test-retest using 4 different variables to express hypoxic ventilatory response and 2 different variables to express hypercapnic ventilatory response.

Variable	Number of Tests	R value	P value
**Hypoxia-all** = (V_e_ at maximal hypoxia–V_e_ at baseline)/(SpO_2_ at baseline—SpO_2_ at maximal hypoxia)	28	0.499	0.007
**Hypoxia-90** = (V_e_ at maximal hypoxia–V_e_ at 90%SpO_2_)/ (90—SpO_2_ at maximal hypoxemia)	28	0.389	0.041
**Regression**_**(Hypoxia-all)**_ = Slope of the regression line of V_e_ on SpO_2_ (all measurements)	28	0.400	0.035
**Regression**_**(Hypoxia-90**_**)** = Slope of the regression line of V_e_ on SpO_2_ (only measurements done when SpO_2_ **</ =** 90%)	28	0.468	0.012
**Hypercapnia-all** = (V_e_ at maximal hypercapnia–V_e_ at baseline)/(EtCO_2_ at maximal hypercapnia—EtCO_2_ at baseline)	16	0.668	0.005
**Regression**_**(Hypercapnia -all)**_ = Slope of the regression line of V_e_ on SpO_2_	16	0.509	0.044

### Marker selection and genotyping of individual polymorphisms

In addition to the respiratory control tests, participants provided a blood sample for HLA DQB1*06:02 typing and for genome wide association (GWAS) analysis. HLA-DQB1*06:02 positivity was determined by PCR amplification using codon 9 and codon 30 sequence-specific primers (plus DR generic primers to control for the presence of amplicon, resulting in the amplification of a control band). In addition, whole genome genotyping was done in all Caucasians using the Affymetrix 6.0 platform (Affymetrix, CA), N = 199. The goal was not to conduct a genome wide association study, as the sample is too small, but to obtain genotypes covering a few selected loci known to be associated with central ventilatory control.

### Genome analysis

Markers with missingness less than 5% at the genotype and individual level were kept in the analysis. Imputation of selected markers was performed by first phasing the genotypes with SHAPEIT [10] and using IMPUTE2 for imputation [11] and 1000 genomes phase I integrated haplotypes. The SNPs for PHOX2B, GPR4, and KCNK5/TASK2 were selected based on Hapmap2 tagSNP picker. TagSNPs were selected to cover the variation in the coding region (rs6850846, rs6831893 and rs11730903) and in an evolutionary conserved region 18 kb upstream from the transcription start site (rs1540499, rs4861145, rs4861011). All tested variants passed quality control and had imputation quality over 0.8.

### Statistics

The hypothesis to be tested was that healthy individuals with a mutation in the HLA- DQB1*06:02 allele would have a reduced HVR. We used IBM SPSS Statistics for Windows, Version 21.0 (IBM, Armonk, NY 2012). For comparison of the continuous variables we used t-tests (for normal distributed variables) and Mann–Whitney U tests (in case of non-normal distribution). Chi-square tests were used for categorical variables. For correlations we used the Pearson correlation coefficient (for normal distributed variables) or the Spearman's rank correlation coefficient tests (in case of non-normal distribution). Analysis of variance (ANOVA) was used to determine the contribution, if any, of variables such as age, BSA, and gender on HVR and HCVR. BSA was calculated using the Mosteller formula. Since ANOVA assumes normal distributed variables and since the two major variables, namely HVR and HCVR, were skewed, we first ln-transformed each of these variables into a new variable normally distributed in the form of ln(HVR/HCVR +1). The addition of 1 to the measured variable was done in order to assure the positivity of the number in the brackets. A p-value <0.05 was considered significant. For other genetic variants the analyses were done with SNPTEST v2.5 using a frequentist association model. These analyses were considered exploratory and not corrected for multiple comparisons.

## Results

### Baseline breathing parameters

Comparison of baseline breathing parameters between the genders (Table 1) reveals that women had higher baseline respiratory rate but lower baseline minute ventilation than men, and these findings remained significant after correcting for BSA. We also found that baseline oxygen saturation (SpO2) was higher in women; whereas, baseline exhaled CO2 (EtCO2) was higher in men. These changes sustained correction for age, ethnicity and BMI. No differences were seen in the age or ethnicity between the sexes.

### Calculation of hypoxic and hypercapnic ventilatory response—test retest analysis

**Fig 3:**
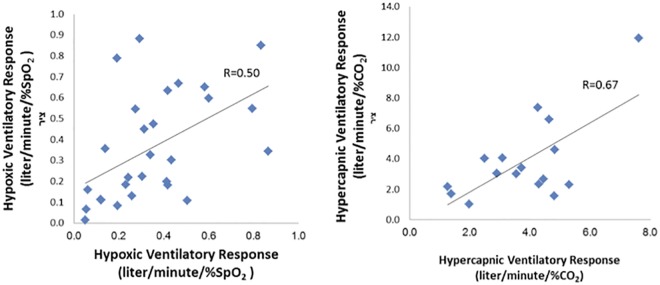
Correlation between test and re-test of hypoxic (N = 28 participants) and hypercapnic (N = 16 participants) responses.

Two repeated hypoxia and hypercapnia response tests were done on 16 individuals and then on 28 individuals. "Hypoxia-all" and "Hypercapnia-all" were the variables with the highest correlation between paired studies (Table 2) and were used throughout the study to express HVR and HCVR. Of note, we found stronger concordance in the paired measurements of hypercapnia than for hypoxia (R = 0.67 and R = 0.50, respectively). See Fig 3 and Table 2.

### Epidemiologic and physiologic variables influencing hypoxic ventilatory response (Table 3)

We assessed the influence of age, gender, ethnicity, BSA, and the level of EtCO2 at the end of the procedure on the response to hypoxia. Of these 5 variables, gender, BSAand the level of EtCO2 at the end of the study were found to be significantly associated with the level of response to hypoxia. The strongest influence on HVR was gender. In women mean HVR was 0.276±0.168 (liter/minute/%SpO2) compared to 0.429±0.266 (liter/minute/% SpO2) in men, p<0.001. This represents a 55.4% higher ventilatory response to hypoxia in men. After controlling for differing BSA between genders, the difference in HVR attenuated but remained significant: HVR/BSA was 0.16±0.10 (liter/minute/%SpO2/m2) in women vs.0.22±0.13 (liter/minute/% / m2) in men (p<0.001). This represents a 37.5% higher ventilatory response to hypoxia in men. Since women had lower baseline minute ventilation (BLMV) we also compared the response to hypoxia between genders after controlling for BLMV. After this correction the difference in HVR became borderline; HVR/BLMV was 0.046± 0.035 (liter/minute/% SpO2)/(liter/minute) in men and 0.040±0.042 (liter/minute/% SpO2)/(liter/minute) in women, p = 0.055.

**Table 3:** Hypoxic (HVR) and hypercapnic (HCVR) ventilatory responses in males vs females.

	Males	Females	P-value
HVR (liter/minute/%SpO_2_)	0.43±0.27 (n = 170)	0.28±0.17 (n = 251)	<0.001
HVR/BSA (liter/minute/%SpO_2_/m^2^)	0.22±0.131 (n = 169)	0.16±0.10 (n = 249)	<0.001
HVR/BLMV (liter/minute/% SpO2)/(liter/minute)	0.046± 0.035 (n = 169)	0.040±0.042 (n = 251)	0.055
HCVR (liter/minute/%co2)	3.30±1.76 (n = 125)	2.89±1.41 (n = 236)	0.045
HCVR/BSA (liter/minute/%CO_2_/m^2^)	1.74±1.06 (n = 125)	1.71±0.87 (n = 233)	0.713
HCVR/BLMV (liter/minute/% SpO2)/(liter/minute)	0.368±0.335 (n = 125)	0.497±1.61 (n = 236)	0.178

Some of the data is not available for all participants. The number of participant for whom the data exists is written in brackets.

Since HCVR is associated with HVR (see below), we reassessed the relation between gender and HVR with correction to HCVR. Adding HCVR to the aforementioned variants (gender, BSA, length of the study and the level of EtCO2 at the end of the study) did not change the significance of the association between HVR and gender (P = 0.011).

A statistically significant but weak positive correlation was found between BSA and HVR (R = 0.2885, p<0.001) and between EtCO2 at the end of the study and HVR (R = 0.124, p = 0.011). No correlation was found between HVR and ethnicity (R = - 0.008) or age (R = - 0.026). In ANOVA, in which HVR was a dependent variable and gender, BSA, and EtCO2 at the end of the study were variants, only gender and BSA remained significant (p<0.001, p = 0.015, respectively). HVR and SpO2 were not significantly correlated (r = -0.109 and r = 0.033 in males and females respectively, n.s.).

### Epidemiologic and physiologic variables influencing hypercapnic ventilatory response

We assessed the influence of age, gender, origin, and BSA on HCVR. Of these variables, only gender had an association with HCVR. As was found with hypoxia, men’s response to hypercapnia was higher. The extent of the difference was lower at 14.2% (3.30±1.76 liter/minute/%CO2 for men vs 2.89±1.41 liter/minute/% CO2 for women, p = 0.045), and the association did not sustain with correction for BSA (p = 0.507) or baseline minute ventilation (p = 0.178). Age, ethnicity, and BSA did not associate with HCVR (p = 0.29 for age, 0.38 for ethnicity, and p = 0.321 for BSA).Some of the data is not available for all participants. The number of participant for whom the data exists is written in brackets.

### Hypoxic ventilatory response correlates with hypercapnic ventilatory response

**Fig 4:**
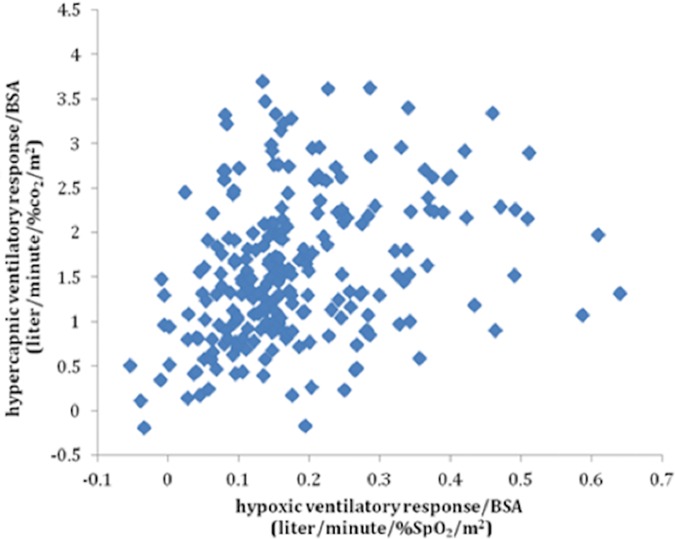
Association between Hypoxic and Hypercapnic Ventilatory Responses. R = 0.392, p<0.001.

We calculated the correlation between HVR and HCVR in the 299 participants who performed both tests. There was a positive correlation between the two (R = 0.392, p<0.01, (Fig 4). The correlation remained significant after correction for gender and BSA (p<0.01).

### HLA-DQB1*06:02 does not associate with response to hypoxia or hypercapnia

No significant correlation was found between the presence of HLA-DQB1*06:02 and HVR. Mean HVR was 0.334± 0.216 (liter/minute/%SpO2) in participants who were HLA-positive and 0.338±0.229 (liter/minute/%SpO2) in participants who were HLA-negative (p = 0.985). Since the association between DQB1*06:02 and HVR was reported in a Chinese population (3), we assessed the association between HVR and HLA-DQB1*06:02 in the Asian subgroup of our participants (98 individuals). Only 5 were HLA-positive. In this group there was no association between HLA and HVR. Mean HVR was 0.307±0.204 (liter/minute/%SpO2) in HLA-negative participants and 0.342±0.201 (liter/minute/%SpO2) in HLA-positive participants (p = 0.70). Similar results were seen after correction for BSA. Nor was an association seen between HLA-DQB1*06:02 and HCVR. Mean HCVR was 3.24 ± 1.66 liter/minute/% CO2 in HLA-positive participants and 2.96 ± 1.52 liter/minute/% CO2 in HLA-negative participants (p = 0.233).

### Association of genetic variants with the response to hypoxia and hypercapnia

We also investigated the influence of SNPs in the PHOX2B, GPR4 and TASK2/KCNK5 genes on HVR and HCVR using tagSNP approach and included tagging SNPs within 5kb up and downstream of the genes (S1 Table). In addition, coding polymorphisms were studied. Because these associations were not corrected for multiple comparisons, none are considered statistically significant. The only suggestive association was found for HCVR with TASK2/KCNK5 rs2815118 (intronic tagging SNP; p = 0.0378, β = 0.261, SE = 0.124) and rs150380866 (coding variant Arg->Trp, p = 0.0284, β = 1.973, SE = 0.892).

## Discussion

### Main findings

The primary goal of this study was to evaluate a previous finding of reduced HVR in carriers of HLA DQB1*06:02 [3]. We did not confirm this observation. We also did not find a relation between the previously implicated genes and HVR or HCVR. Nonetheless, the large number of healthy individuals in our study enabled us to assess the association between epidemiological characteristics and control of breathing. We noted a strong association between gender and HVR and likely no association between gender and HCVR.

### HLA DQB1*06:02 and hypoxic ventilatory response

HLA DQB1*06:02 and hypoxic ventilatory response were not associated with HVR in our population, in contrast to Han’s observation in Chinese population [3]. As DQB1*06:02 was common in our sample, we had plenty of power to replicate this initial finding. One explanatory mechanism could be ethnic-specific linkage between DQB1*06:02 and a variant gene for HVR that has yet to be elucidated. The potential variant gene could dictate lower HVR compared to other variant(s). Our study included a spectrum of ethnic groups, but only 5 of the 98 Asian participants were positive for HLA-DQB1*06:02. We thus have limited ability to draw conclusions about the relationship between HLA and HVR in an Asian population.

### SNPs in the PHOX2B, GPR4 and TASK2/KCNK5 genes and ventilatory responses

More than 90% of patients with Congenital Central Hypoventilation Syndrome (CCHS) have been found to have a mutation in the PHOX2B gene [12]. We did not find a relationship between 6 different SNPs of the PHOX2B gene and HVR or HCVR in healthy volunteers. The clinical distinction between this finding in our awake participants and the importance of PHOX2B in CCHS is not entirely surprising, given that voluntary control of breathing is markedly diminished during sleep.

Most recently, RTN neurons in mice were found to detect CO2 via two intrinsic proton receptors (TASK-2/ KCNK5, GPR4) (7,8). We found point wise significant effects between two TASK2/KCNK5 variants (rs2815118 and rs150380866) and HCVR. Association with rs150380866 results in a synonymous mutation in Arg263Trp that, although rare (allele frequency~0.0023) and not in a known protein motif, could well have functional effects.

### Gender and ventilatory responses

Our major positive finding of the study is an HVR that is 55.4% higher in men. After controlling for BSA, the difference decreases but remains high (31.2%). The difference became borderline after correction to baseline minute ventilation, which is lower in women. The small difference between genders for HCVR is fully explained by gender difference in BSA. Previous reports about the influence of gender on HVR and HCVR show conflicting results. Somestudies showed a lower HVR in men [13,14]. Other studies found an HVR that was greater in men compared to women, with a range of 39–110% [15,16,17]. There also are data showing no gender difference [18,19, 20]. Prior studies evaluated between 11 to 67 subjects in total, in comparison to the 427 participants with reliable data in our study. To our knowledge, it is the largest cohort to date. The higher HVR response observed in men is interesting, as it could contribute to higher sleep disordered breathing in men versus women by promoting instability of central respiratory control feedback loops [21–24].

Multiple mechanisms could account for gender difference in HVR. The difference may be secondary to progesterone’s effect as a respiratory stimulant. Progesterone levels are higher in women through all phases of the menstrual cycle [25, 26], and peripheral chemoreceptors may be sensitive to progesterone levels [27,28]. Other effects may also be involved. For example, a study in mice has shown lower HVR in males, an effect opposite than what we observed here and mediated by erythropoietin [29]. Another possibility is that the anatomical differences of the respiratory system between genders require normalizing for vital capacity and FEV1 to attenuate HVR [16]. We did not perform lung function studies; however, the finding that the difference in HVR between genders becomes borderline after correction to baseline minute ventilation suggests that the difference between genders might be related to anatomical differences of the respiratory system, which might be more efficient in females. Finally, although this population was mostly comprised of healthy young individuals, men had higher BMI. Men are more prone to sleep apnea, and some individuals could have undiagnosed sleep disordered breathing. Exposure to intermittent hypoxia and sleep apnea may have exacerbated and increased their HVR with the passage of time. Intermittent hypoxia has been shown generally to increase sensitivity to hypoxia through long-term facilitation in animals and humans [30], although it is unclear if the effect persists during long-term, chronic intermittent hypoxia as observed in sleep apnea [30].

The literature concerning the influence of gender on human HCVR also is not homogeneous. Some studies found a higher HCVR in men [1,15,16, 31]. Two of the studies found that this difference resolved after adjustment for FEV1 [16, 31]. Hirshman and colleagues [18] found no difference between genders. While men in our study have an HCVR that is 14.2% higher than women, the difference disappears after adjustment for BSA. We infer that there is no gender difference in the response to hypercapnia.

Sex differences in the control of breathing are not unique to humans and have been reported in mammals in response to various stimuli. For example, aspartic acid depresses ventilation in awake male but not female rats [32] and adult male (but not female) rats subjected to neonatal caffeine treatment show a higher breathing frequency response during the early phase of hypoxic exposure [33]. Similarly, adult male rats previously subjected to a stress have an abnormally elevated hypoxic ventilatory response, and female rats do not [34]. Further, animal studies clearly demonstrate complex and multigenic genetic control for these traits [3,35,36]. Although our study demonstrates the feasibility of conducting genetic association for these traits, future studies with a vastly increased sample size will be needed to identify genetic factors with higher statistical confidence.

### Gender differences in Ve, EtCO2, and SpO2

Women had higher SpO2 and lower EtCO2 compared to men (98.0±1.3% vs. 96.6±1.7% and 4.65±0.68 vs. 4.82±1.02, respectively), which they achieved despite lower baseline minute ventilation (8.08±2.36 l/m vs. 10.00±3.43l/m). Lower Ve in women has been reported before [13], although in these stud the difference attenuated to zero with correction for BSA. In other studies with similar findings, Ve was not corrected for BSA [37] or the difference in Ve was not statistically significant [5]. Our study is significantly larger than prior investigations, and the difference in Ve from our data remained significant even after correction for BSA.

Lower EtCO2 in women has been found previously [15,13]. Some investigators note that the difference becomes statistically significant during the follicular phase of the menstrual cycle [20].

Although gender differences in SpO2 have been seen in studies assessing transcutaneous PaO2 [38], we could find only one study that pursued this query as its main goal [39]. Ricart and colleagues found women to have a higher baseline SpO2 at rest that is statistically significant. van Klaveren [16] describes a similar difference, but no test for statistical significance was performed. Differences in hemoglobin affinity for oxygen and in oxygen transport have been reported between sex and could be involved [40,41], as could the stimulating effects of progesterone on breathing [27, 28].

The 1.4% higher Sp02 in women versus men may have small clinical implications when large groups of subjects are studied. When the body experiences hypoxia due to chronic disease, such as sleep apnea, obesity hypoventilation, or COPD, small decrements of oxygen could have significant consequences over years of injury. For patients in extremis, modest differences in arterial oxygen may profoundly affect morbidity and mortality. Indeed, higher mortality is seen in men compared to women for Acute Respiratory Distress Syndrome (ARDS) [42]. Oxygenation is a complex challenge in such a context, as it requires balance between achieving adequate end-organ delivery of oxygen and minimizing presumed damage from free radicals.

### Relationship between responses to hypoxia versus hypercapnia

Our results suggest that individuals with a higher response to hypoxia also have a higher response to hypercapnia (R = 0.34), a phenomenon has been described before [14,18,43]. One explanation may arise from the observation that neural output from peripheral chemoreceptors increases in the presence of hypercapnia [44,45]. An alternative mechanism could be related to changing levels of O2 and CO2 as a consequence of respiratory mechanics, in which case correction to FEV1 may change the magnitude of correlation.

### Accuracy of tests

The tests for HVR and the HCVR commonly used for assessment of control of breathing have low reproducibility within and between individuals [46]. In order to increase accuracy we conducted a preliminary test-retest study to determine the most reproducible method for calculation of HVR and HCVR. The variables hypoxia-all and hypercapnia-all were found to have the best intra-individual correlation in repeated testing and were used to report test results. Given that prior studies applied regression analysis to report HVR and HCVR, we repeated our analysis with all other variables: Hypoxia-90, Regression(Hypoxia-all), Regression(Hypoxia-90) for HVR and Regression(hypercapnia) for HCVR. We found the same results in that an increase in HVR is associated only with gender and not with HLA-DQB1*06:02.

### Study limitations

The current study has several limitations. First, the tests for HVR and HCVR have moderate to low reproducibility. We used two strategies to increase accuracy but acknowledge that high variability between measurements may prevent detection of differences of small magnitude. Second, we had a significant amount of patient traces that could not be included, most notably when testing response to hypoxia during rebreathing. This was mostly related to the length of the hypoxia test increasing the possibility of leaks and claustrophobia. Third, pulmonary function testing, which was not performed in the current study, might have been an additional way to account for the difference in gender and in body mass between the participants of the study. Fourth, the study might not be powered to detect a low impact of the HLA on HVR. Finally, we did not collect information concerning the menstrual cycle and cannot comment on its influence on the ventilatory responses of women. Given that progesterone is a known respiratory stimulant, one can reasonably conclude that progesterone level does not account for our finding of decreased HVR in women. We also cannot comment on the effect of hormonal differences on arterial oxygen or oxygen delivery. Studies have shown that women who are post-menarche and pre-menopause have less hemoglobin affinity for oxygen when compared to men [35].

### Conclusion

In summary, we studied HVR and HCVR in several hundred subjects and found higher HVR but no difference in HCVR for men versus women. Women also had lower baseline minute ventilation, higher SpO2, and lower EtCO2. No association was seen between HLA-DQB1*06:02 and HVR or HCVR. Genetic analysis revealed point wise, uncorrected significant associations between two TASK2/KCNK5 variants (rs2815118 and rs150380866) and HCVR, but these exploratory findings would need replication. The studies demonstrate the feasibility of conducting large scale ventilation studies for genetic analysis, such as Genome wide Association studies.

## Supporting Information

S1 Table this table shows the influence of SNPs in the PHOX2B, GPR4 and TASK2/KCNK5 genes on HVR and HCVR using a tagSNP approach including tagging SNPs within 5kb up and downstream of the genes. (XLSX)

S1 File Raw data used in the analysis. (XLS)
